# Dose of Chloral

**Published:** 1871-09

**Authors:** 


### Dose of Chloral.

Dr. Richardson (*Pharmaceutical Trans.*) believes that it is not prudent to administer more than 120 grains of chloral-hydrate in 24 hours, and that 180 grains is a fatal dose, 120 a dangerous one. [50 grains is a dangerous dose under ordinary circumstances.—Ed.] —*New Remedies.*

